# Clinical Outcomes After Stereotactic Body Radiation Therapy for Early Stage Non-Small Cell Lung Cancer: A Single Institutional Study

**DOI:** 10.7759/cureus.11886

**Published:** 2020-12-03

**Authors:** Michael Abdalmassih, Oliver Bucher, Shrinivas Rathod, Arbind Dubey, Julian O Kim, Naseer Ahmed, Ahmet Leylek, Amitava Chowdhury, Bashir Bashir

**Affiliations:** 1 Family Medicine, University of Manitoba, Winnipeg, CAN; 2 Department of Epidemiology and Cancer Registry, CancerCare Manitoba, Winnipeg, CAN; 3 Department of Radiation Oncology, CancerCare Manitoba, University of Manitoba, Winnipeg, CAN

**Keywords:** stereotactic radiotherapy, sbrt, lung cancer, nsclc

## Abstract

Introduction

The standard of care for early-stage non-small cell lung cancer (NSCLC) is surgery. However, for medical inoperable patients stereotactic body radiation therapy (SBRT) is an alternative method. The aim of the study is to assess the overall survival (OS), progression-free survival (PFS) and local control (LC) of patients diagnosed with NSCLC in Manitoba, Canada, between 2013 and 2017 and managed with SBRT.

Materials and methods

This retrospective study included a total of 158 patients (60.13% of the population were females) that were diagnosed with stage I-II NSCLC and were treated with lung SBRT between 2013 and 2017 in Manitoba. Demographics and clinical data were retrospectively extracted from the electronic patient record. Kaplan-Meier and Cumulative incidence curves were used to describe the OS, PFS, and LC outcomes.

Results

From the 158 patients, 32 patients were treated with 60 Gy in eight fractions, while 121 patients were treated with 48 Gy in four fractions. Only 85 patients had biopsy-proven NSCLC. The median OS was 2.87 years (95% confidence interval [CI] 2.16-3.43). OS rates at one and two years were 85% and 66%, respectively. The median PFS was 2.03 years (95% CI 1.65-2.77). Furthermore, one-year and two-year PFS rates were 77% and 51%, respectively. Only 10 patients progressed locally at one year and 17 at two years, making the LC rate 93% at the one-year and 87% at the two-year mark.

Conclusion

These findings add to a growing evidence base supporting SBRT in the treatment of clinically suspected and biopsy-proven early-stage NSCLC patients.

## Introduction

The most common malignancy worldwide to date is lung cancer, accounting for 11.6% of all new cancers diagnosed [1,2], and it affects 29,300 patients per year in Canada [3]. Lung cancer remains the leading cause of cancer death among men and women, resulting in 1,761,007 (18.4%) deaths annually worldwide [1,2]. In Canada, during 2019, an estimated 82,000 Canadians were expected to die from cancer, and one in four cancer-related deaths was anticipated due to lung cancer [3]. At an advanced stage, survival is very low; approximately 50% of lung cancers diagnosed in 2017 were stage IV [3]. Lung cancer has many subtypes, but the main subtypes of lung cancer are non-small cell lung cancer (NSCLC) and small cell lung cancer (SCLC). The most common form of lung cancer is NSCLC, accounting for more than 80% of all lung cancers and stage IV NSCLC was approximately half of all diagnosed lung cancer in 2017 in Canada compared to only one-fourth (23.1%) being at stage I and 8.9% being at stage II [3]. In the province of Manitoba, an average of 2,797 patients are diagnosed with stage I NSCLCs and an average of 1,106 patients with stage II annually, accounting for 24.6% and 9.5% of NSCLCs, respectively [3].

There is no screening program for the early detection of lung cancer in Canada although there are studies investigating the feasibility of low-dose computed tomography (CT) for high-risk populations [4]. Chest CT screening for high-risk patients plays a valuable role in the early detection of lung cancer, and patients with early-stage lung cancer have a reasonably high chance of local control [5]. The most significant single risk factor for being diagnosed with lung cancer is smoking, responsible for 90% of lung cancer in men and 80% of lung cancer in women. Furthermore, exposure to atmospheric pollutants such as asbestosis adds to the individual risk of lung cancer [6].

A tissue diagnosis is usually made before commencing radiotherapy; however, some patients may have significant comorbidities that may limit tissue diagnosis by biopsy [7]. In such cases, solitary pulmonary nodule remains a diagnostic challenge, and a diagnostic algorithm could be used to validate a lesion to be more or less malignant [8]. A fluorodeoxyglucose (FDG) positron emission tomography (PET) CT scan is indicated for all patients with suspected early-stage NSCLC to rule out distant metastasis. Pulmonary function tests are also conducted to ensure adequate lung function [9].

For clinically operable early-stage NSCLC, surgical resection is the best treatment modality to achieve a cure [10]. For those patients who are inoperable, stereotactic body radiotherapy (SBRT) has been an alternative that provides excellent local control (LC) [11,12]. SBRT is unlike conventional radiotherapy, whereby high ablative doses of radiotherapy is delivered with high precision and steep dose gradients, maximizing dose to the growth tumor volume and minimizing harm to healthy surrounding tissues [9]. The treatment planning with tight margins, image guidance, and strict immobilization allows the delivery of high biologically effective dose (BED) to the growth tumor sparing organs at risk. With this approach, acute organ toxicities are limited, and deterioration of quality of life is not common [13]. Pneumonitis and chest wall toxicity, including skin reactions, chest wall pain, and rib fractures, are the most common reported toxicities but are seen at clinically acceptable levels.

In Manitoba, SBRT has been offered to medically inoperable patients in early-stage NSCLC patients since 2013. Not all patients who were treated with SBRT had tissue confirmation for malignancy. Furthermore, in this study, we analyze the clinical outcome of patients who were treated with SBRT, whether they had a biopsy or not confirming malignancy in Manitoba.

## Materials and methods

Study population and data accrual

This retrospective chart review was conducted on patients diagnosed with early-stage (stage I-II) non-small cell lung cancer in the province of Manitoba, Canada, between January 2013 and December 2017. Patients were included in the study whether they were diagnosed clinically or through biopsy confirming NSCLC. The study was approved by the regulatory local ethics committee. Patients were identified through the radiotherapy treatment record registry.

Initial tumor node metastasis staging was determined based on imaging studies, including computed tomography of chest, abdomen, and pelvis, PET-CT scans. The overall staging was determined according to the American Joint Committee on Cancer, Seventh Edition guidelines. Local eligibility criteria to receive SBRT included tumors ≤5 cm with or without chest wall involvement; patients with more than one primary lung tumor were also eligible. Patients were deemed medically inoperable or declined surgery. Patients were to be considered N0 if the hilar or mediastinal lymph nodes were ≤ 1 cm and there was no abnormal uptake on FDG PET-CT scan. Patients were excluded from receiving SBRT if organ at risk constraints were not met, the patient was unable to lie flat for 60 minutes, or intolerant of the immobilization device. Two SBRT dose regimes were mainly used. A dose of 48 Gy in four fractions (BED = 106 Gy10), with fractions at least 36 hours apart, was used when the tumor was more than 10 mm away from mediastinal or pericardial pleura for added safety. The other regimen used was 60 Gy in four fractions (BED = 105 Gy10). This dose fractionation was considered if the tumor was located between 6 mm and 10 mm away from mediastinal or pericardial pleura, or dose constraints for four fractions were not met, excluding the chest wall and ribs.

Data collected included patient demographics, staging, pathological details, disease progression, site of progression, and follow up. Data collection was completed on June 1, 2019.

Statistical analyses

Clinical and demographic characteristics of the cohort were described using frequencies and percent for characteristics measured on a categorical scale, while median and interquartile ranges were used to describe characteristics measured on a continuous scale. Measures of overall survival (OS) and progression-free survival (PFS) were estimated using the Kaplan-Meier method, while measures of LC were estimated using cumulative incidence curves. Differences in OS and PFS between categorical patient characteristics were investigated using Log-rank testing, while differences in LC between categorical patient characteristics were investigated using K-sample tests. P values ≤ .05 were considered indicative of a significant difference. Univariable Cox regression models fitted with restricted cubic splines were used to investigate the relationship between non-linear continuous patient characteristics and OS and PFS. Likewise, univariable competing risk models fitted with restricted cubic splines were used to investigate the relationship between LC and non-linear continuous patient characteristics. The number of knots in each spline function was determined using Akaike’s information criteria. Knots were placed at fixed quantiles of the predictor variable’s marginal distribution for each [14].

## Results

A total of 158 patients were included who met the inclusion criteria with a median age of 76 years and an interquartile range of 13. Two-thirds of the study cohort (n=95) were women and one-third were men (n=63). Eighty-five patients (53.8%) had tissue-confirmed NSCLC diagnosis, while 73 (46.2%) did not have a biopsy and were diagnosed clinically. In biopsy-proven NSCLCs, adenocarcinoma was found in 49 patients, almost double the squamous cell carcinoma pathology, which was found in 25 patients. Three-quarters of the study cohort received 48 Gy in four fractions (BED = 106 Gy10). Eighty percent of the population (n=127) were smokers (Table 1).

**Table 1:** Descriptive characteristics of the study cohort (n=158). a – Includes, carcinoma, adenosquamous, and non-small cell lung cancer not otherwise specified.

Characteristic	Frequency	Percent
Sex		
Female	95	60.13
Male	63	39.87
Biopsy status		
Yes	85	53.80
None	73	46.20
Cancer type		
Adenocarcinoma	49	31.01
Squamous cell carcinoma	25	15.82
Other^a^	10	6.33
Unknown	74	46.84
Smoking status		
Smoker	127	80.38
Non-smoker	6	3.80
Unknown	25	15.82
Number of fractions		
3	1	0.63
4	121	76.58
5	3	1.90
8	32	20.25
15	1	0.63

**Figure 1:**
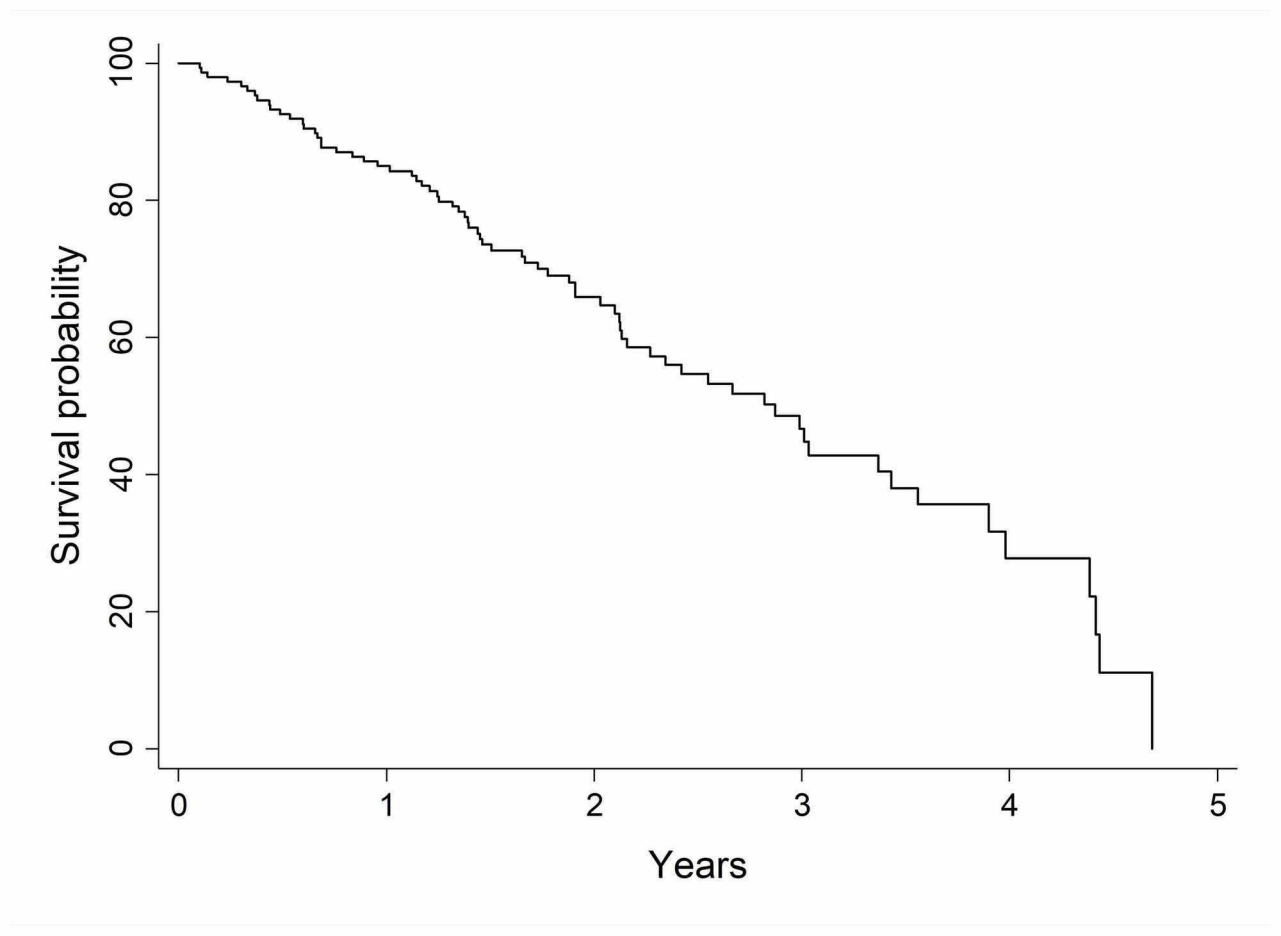
Kaplan-Meier curve of overall survival for the whole study cohort (n = 158)

The median OS time was 2.87 years (range, 2.16 to 3.43 years). One- and two-year OS were found to be 85% and 66%, respectively (Figure 1). When compared to men, women had statistically superior OS (P-value = .02) with 92% (95% CI 0.84-0.96) and 75% (95% CI 0.63-0.83) at one year and two year compared to 74% (95% CI 0.61-0.83) and 52% (95% CI 0.38-0.65). Smoking, biopsy status, pathology type, and tumor size did not have an impact on the OS in this cohort (Table 2).

**Table 2:** Kaplan-Meier overall survival analysis for the study cohort (n= 158) Abbreviations: CI, confidence interval; N/A, not applicable. *The formula for CI calculation addresses the presence of events. Therefore, the upper CI limit for these events could not be estimated for the latter portion of the survival curve where fewer subjects are at risk.

Variables	Year	Deaths	Median survival (95% CI)	Survivor Function (95% CI)	P value
Sex					.02
Female	Overall	36	3.01 (2.67-3.98)	-	
	1	7	-	0.92 (0.84-0.96)	
	2	12	-	0.75 (0.63-0.83)	
Male	Overall	34	2.03 (1.35-3.37)	-	
	1	15	-	0.74 (0.61-0.83)	
	2	11	-	0.52 (0.38-0.65)	
Smoking status					
Non-smoker	Overall	1	N/A	-	.10
	1	0	-	1.00	
	2	0	-	1.00	
Unknown	Overall	10	2.87 (2.12-…)*	-	
	1	2	-	0.92 (0.71-0.98)	
	2	2	-	0.80 (0.55-0.92)	
Smoker	Overall	59	2.82 (2.03-3.43)	-	
	1	20	-	0.83 (0.75-0.89)	
	2	21	-	0.61 (0.51-0.70)	
Biopsy status					
None	Overall	33	2.67 (2.13-3.03)	-	.40
	1	12	-	0.83 (0.72-0.90)	
	2	11	-	0.64 (0.51-0.75)	
Biopsied	Overall	37	3.37 (2.03-4.41)	-	
	1	10	-	0.87 (0.77-0.93)	
	2	12	-	0.67 (0.55-0.77)	
Pathology status					
Unknown	Overall	35	3.37 (1.91-4.41)	-	.90
	1	12	-	0.83 (0.73-0.90)	
	2	12	-	0.63 (0.49-0.74)	
Other	Overall	4	2.67 (0.38-…)*	-	
	1	2	-	0.75 (0.31-0.93)	
	2	1	-	0.63 (0.23-0.86)	
Adenocarcinoma	Overall	21	2.34 (1.73-…)*	-		
	1	5	-	0.89 (0.75-0.95)	
	2	9	-	0.63 (0.45-0.76)	
Squamous	Overall	10	2.99 (2.10-3.03)	-	
	1	3	-	0.86 (0.63-0.95)	
	2	1	-	0.82 (0.58-0.93)	

**Figure 2:**
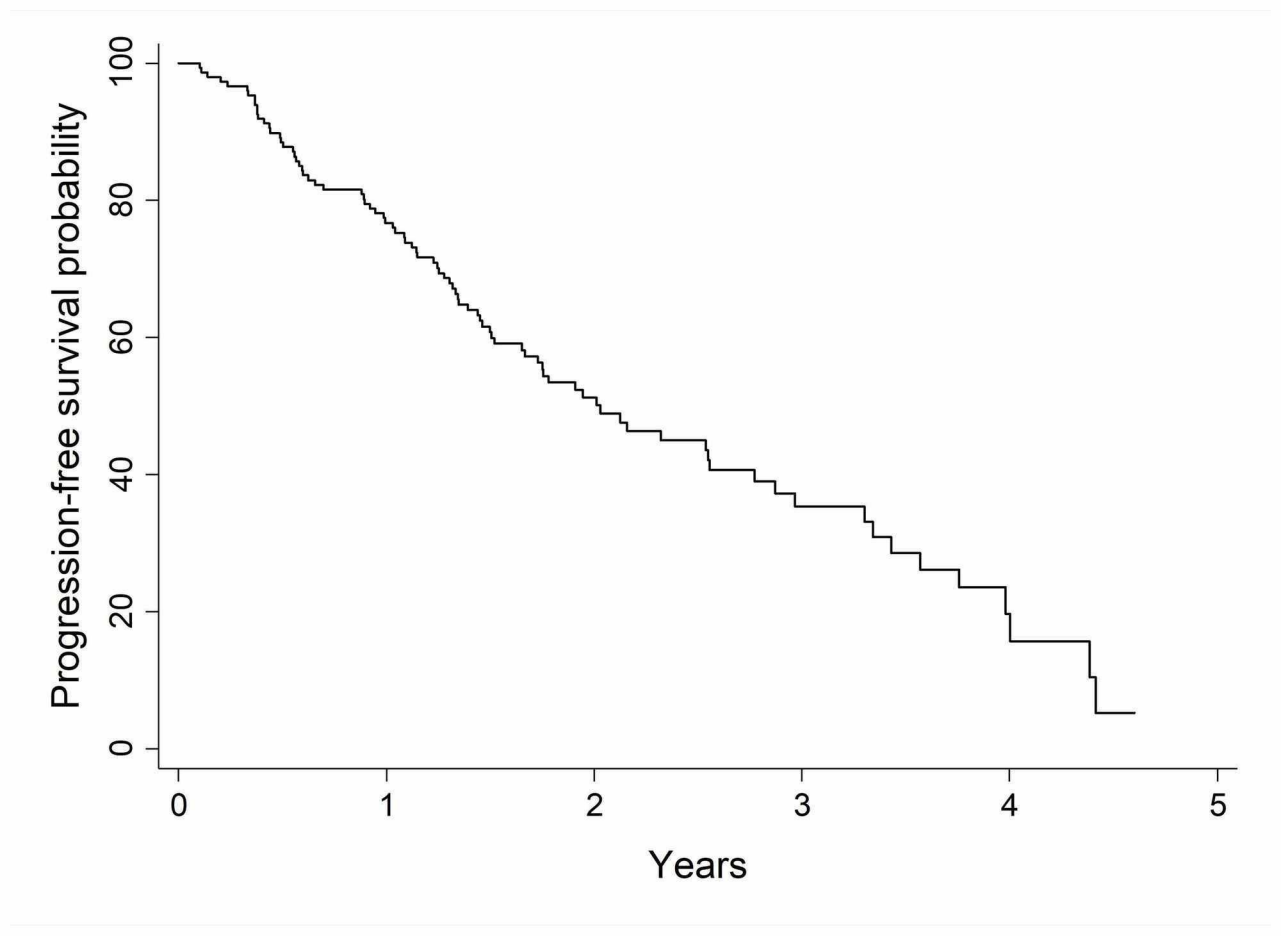
Kaplan-Meier curve of progression-free survival for the whole study cohort (n = 158)

The overall median PFS of the cohort was 2.03 years (range, 1.65-2.77). At one year, the progression-free survival rate was 77% (95% CI 0.69-0.83) and at two years was 51% (95% CI 0.42-0.60) (Figure 2). Although sex was not significant in terms of PFS, women had a higher PFS (P-value = .11), with 82% (95% CI 0.72-0.88) and 59% (95% CI 0.47-0.69) at one year and two years compared to 69% (95% CI 0.55-0.79) and 40% (CI 0.27-0.53) (P-value = .11). Furthermore, smoking, biopsy status, pathology type, and tumor size did not influence the PFS of this cohort (Table 3).

**Table 3:** Kaplan-Meier progression-free survival analysis for the study cohort (n= 158) Abbreviations: CI, confidence interval. *The formula for CI calculation addresses the presence of events. Therefore, the upper CI limit for these events could not be estimated for the latter portion of the survival curve where fewer subjects are at risk.

Variable	Year	Deaths	Median survival (95% CI)	Survivor Function (95% CI)	P value
Sex					.11
Female	Overall	46	2.77 (1.73-3.34)	-	
	1	16	-	0.82 (0.72-0.88)	
	2	17	-	0.59 (0.47-0.69)	
Male	Overall	39	1.67 (1.24-2.12)	-	
	1	18	-	0.69 (0.55-0.79)	
	2	14	-	0.40 (0.27-0.53)	
Smoking status					.14
Non-smoker	Overall	2	3.57 (1.04-…)*	-	
	1	0	-	1.00	
	2	1	-	0.83 (0.27-0.97)	
Unknown	Overall		2.16 (1.75-3.30)	-	
	1		-	0.92 (0.72-0.98)	
	2		-	0.70 (0.44-0.85)	
Smoker	Overall	71	1.75 (1.35-2.55)	-	
	1	32	-	0.73 (0.63-0.80)	
	2	26	-	0.46 (0.36-0.55)	
Biopsy status					.43
None	Overall	41	2.55 (1.50-3.34)	-	
	1	18	-	0.75 (0.63-0.83)	
	2	10	-	0.57 (0.44-0.69)	
Biopsied	Overall	44	1.75 (1.44-2.54)	-	
	1	16	-	0.79 (0.67-0.86)	
	2	21	-	0.46 (0.34-0.58)	
Pathology					.63
Unknown	Overall	42	2.55 (1.50-3.34)	-	
	1	19	-	0.74 (0.62-0.82)	
	2	10	-	0.57 (0.43-0.68)	
Other	Overall	4	2.32 (0.38-…)*	-	
	1	2	-	0.75 (0.31-0.93)	
	2	1	-	0.63 (0.23-0.86)	
Adenocarcinoma	Overall	24	2.03 (1.39-3.57)	-	
	1	7	-	0.84 (0.69-0.92)	
	2	12	-	0.52 (0.35-0.66)	
Squamous	Overall	15	1.52 (0.92-…)*	-	
	1	6	-	0.73 (0.49-0.87)	
	2	8	-	0.34 (0.15-0.54)	

**Figure 3:**
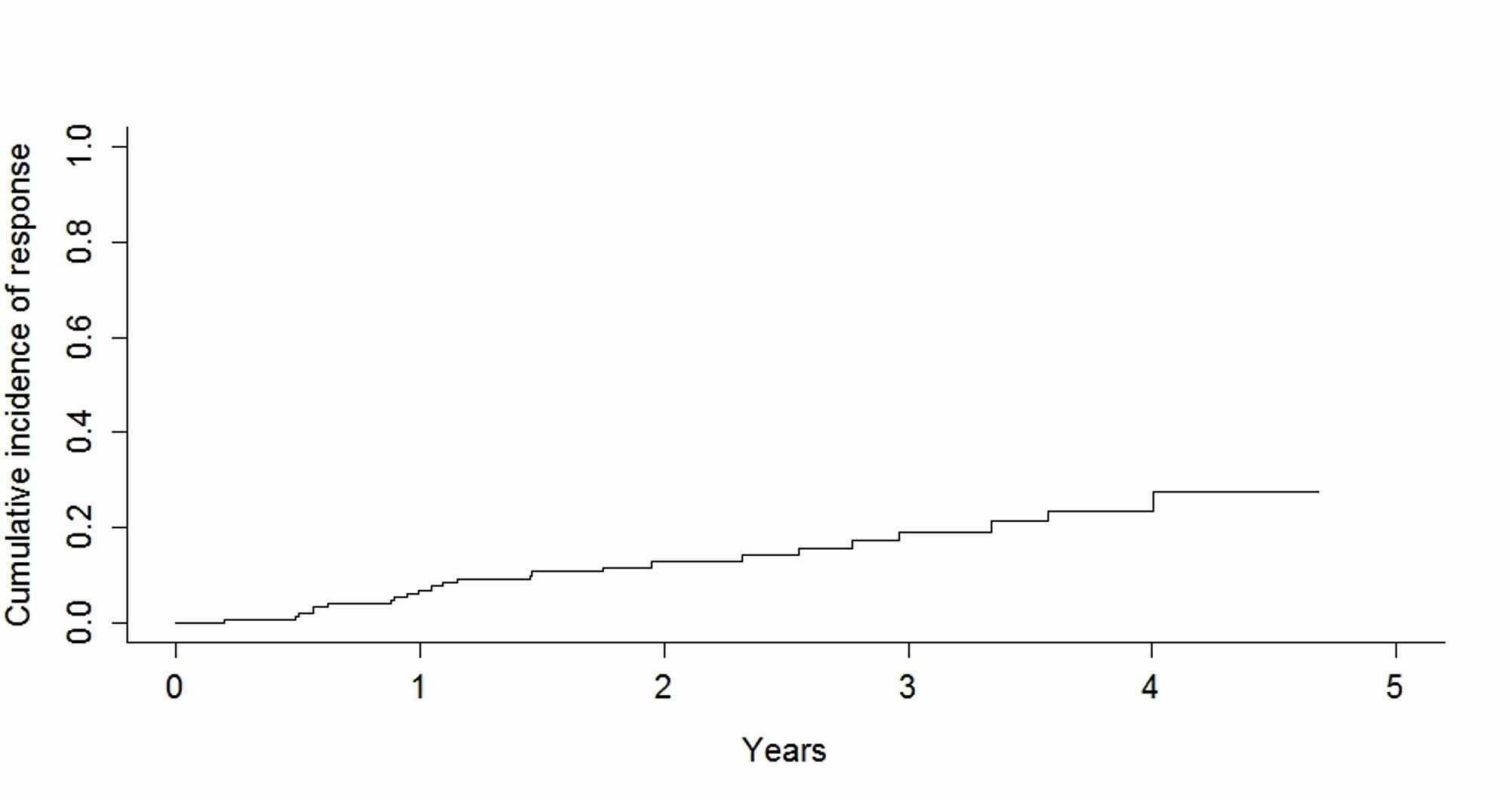
Cumulative incidence curve of local progression for the whole study cohort (n = 158)

The local progression cumulative probabilities at one year and two years were 10 patients accounting for 7% and 17 patients accounting for 13%, respectively (Figure 3). Similarly, smoking, biopsy status, pathology type, and tumor size did not affect the local progression in this population (Table 4).

**Table 4:** Cumulative incidence analysis of local progression for the study cohort (n= 158) Abbreviations: CI, confidence interval. *The formula for CI calculation addresses the presence of events. Therefore, the lower CI limit for these events could not be estimated for the initial portion of the survival curve where fewer subjects are at risk.

Variables	Year	Local progressions	Cumulative incidence Function (95% CI)	P value
Sex				.17
Female	1	7	0.08 (0.02-0.14)	
	2	13	0.16 (0.08-0.24)	
Male	1	3	0.05 (…-0.11)*	
	2	4	0.08 (0.01-0.16)	
Smoking status				.49
Non-smoker	1	0	-	
	2	1	0.17 (…-0.49)*	
Unknown	1	1	0.04 (…-0.13)*	
	2	2	0.10 (…-0.25)*	
Smoker	1	9	0.08 (0.03-0.13)	
	2	14	0.13 (0.07-0.20)	
Biopsy status				.76
None	1	5	0.07 (0.01-0.13)	
	2	8	0.13 (0.04-0.22)	
Biopsied	1	5	0.07 (0.01-0.13)	
	2	9	0.13 (0.05-0.21)	
Pathology				.12
Unknown	1	6	0.09 (0.02-0.15)	
	2	8	0.12 (0.04-0.21)	
Other	1	0	-	
	2	0	-	
Adenocarcinoma	1	1	0.02 (…-0.07)*	
	2	3	0.08 (…-0.16)*	
Squamous	1	3	0.14 (…-0.28)*	
	2	6	0.28 (0.08-0.48)	

## Discussion

The standard of care for operable early-stage NSCLC is surgery. However, a large patient population suffering from NSCLC are medically inoperable for variable reasons [15]. This population may be managed by SBRT as an option that was found to be safe and effective in prospective settings [11,12]. Among medically inoperable patients is a group of patients who may have an insignificant pulmonary reserve. This may lead to avoiding a diagnostic biopsy due to the potential risks of harm or even death [16]. A rising trend is that this population is being managed empirically [7,10,17].

A group in Montréal, Canada, has reviewed the clinical outcomes of 878 patients that had an early-stage NSCLC managed with SBRT. From this cohort of patients, only 131 patients did not have a biopsy-proven stage I NSCLC. Therefore, they were matched with another 131 patients who had a biopsy which confirmed stage I NSCLC. They found no clinically significant differences observed at three years in OS, LC, and local recurrence (LR) [18].

Another multi-institutional study across Canada, the USA, and Europe included data on early-stage NSCLC patients treated with SBRT. A total population of 701 patients was assessed, of whom 67% had tissue confirmation of their tumors [19]. They looked into the OS, disease-free survival (DFS), cause-specific survival, and rates of LR, regional recurrence (RR), and distant metastasis (DM). They reported the three- and five-year outcomes for OS and DFS were 83.8%, 69%, and 60.6%, 45.5%, respectively. They found that LR, RR, and DM rates at three years were 6.4%, 9.3%, and 14.3%. At five years, the LR, RR, and DM rates increased to 10.5%, 14.3%, and 19.7%, respectively. There were no statistically significant differences between the biopsy and no-biopsy cohorts in survival outcomes or recurrences [19].

A population of almost 600 patients with early-stage NSCLC was also studied in the Netherlands, of which 65% of the treated population with SBRT had no biopsy. The outcomes observed showed no difference between biopsied versus non-biopsied patients, with three-year OS and LC rates of 55% and 90%, respectively [10].

An interesting study published by a group from Yale indicated that there is a rising trend observed in the USA in the number of patients undergoing SBRT without biopsy over time. This project identified almost 7000 patients within the National Cancer Database in the USA with stage I NSCLC who received SBRT between 2003 and 2011. The majority of the cohort (95%) had a tissue biopsy before SBRT; however, over time, the number of patients treated without tissue biopsy increased. Being medically inoperable, smaller tumor size, and facility type were the only factors in multivariate analysis associated with odds of SBRT without biopsy [7].

Looking at the results presented in the current study, they resemble those discussed previously, with LC of 93% at the one year and 87% at the two-year point. Furthermore, we expected a low median OS that was 2.87 years. OS rates at one and two years were 85% and 66%, respectively; this could be due to the extensive comorbidities found in this frail population.

We were not able to report any differences in outcome based on the presence or absence of tissue biopsy, which is comparable to the current literature. Near half of our cohort had no tissue confirmation, which is higher than the Yale study data. One reason could be the higher amount of medically inoperable patients that may have a poor general condition and who are incapable of undergoing certain procedures and investigations. Another reason could be the availability of a multidisciplinary team that discusses thoroughly complex cases in our weekly thoracic disease site conference that have a high comfort level to treat patients empirically based on specific imaging criteria.

One of the limitations of this retrospective study is its relatively small sample size. Also, potential selection biases such as poor fitness, personal choice, and other weaknesses were associated with these studies. Looking at the excellent outcomes in the group of patients that had no tissue confirmation and received empiric treatment, we have to question if this was cancer at the time of treatment, especially when we take into consideration that the smaller and less PET avid tumors were more likely present in this group. Moreover, the lack of follow up may influence the OS results. Finally, this work represents a single institutional experience and is retrospective in nature.

## Conclusions

This study adds to the body of literature that early-stage lung cancer patients treated with SBRT have excellent outcomes, whether they had a biopsy-proven NSCLC or were clinically diagnosed. The authors recommend tissue biopsy and pathologic confirmation whenever possible. However, growing literature, including this study, suggests that treating non-pathologically confirmed disease is reasonable. A multidisciplinary team and discussion are crucial and play a remarkable role in the decision making of treating patients without tissue confirmation.
